# Single Visit Apexification Procedure of a Traumatically Injured Tooth with a Novel Bioinductive Material (Biodentine)

**DOI:** 10.5005/jp-journals-10005-1284

**Published:** 2015-04-28

**Authors:** Navroop Kaur Bajwa, Mahesh Madhukar Jingarwar, Anuradha Pathak

**Affiliations:** Medical Officer, Department of Pedodontics and Preventive Dentistry Government Dental College, Patiala, Punjab, India; Junior Resident, Department of Pedodontics and Preventive Dentistry Government Dental College and Hospital, Patiala, Punjab, India; Professor and Head, Department of Pedodontics and Preventive Dentistry Government Dental College and Hospital, Patiala, Punjab, India

**Keywords:** Case report, Single-visit apexification, Biodentine.

## Abstract

Aim of this article is to present a case wherein single visit apexification of a traumatically injured tooth was done with a bioactive material–Biodentine. An injury sustained between the ages of 6 and 14 can adversely affect pulpal health and interrupt root development. In these instances, apexification is generally the preferred treatment. A 10 years old male patient presented with coronal fracture of the left upper central incisor. Clinical and radiographic assessment showed negative pulpal sensibility and arrested apical root development. Artificial apical barrier induction with Biodentine followed by endodontic treatment and prosthetic rehabilitation was decided as the line of treatment. To conclude, this bioactive and biocompatible calcium-based cement can regenerate damaged dental tissues and represents a promising alternative to the conventional multivisit apexification technique.

**Clinical significance:** Biodentine which is a biologically active cement can be an efficient alternative to the conventional apexification materials which were hitherto recommended.

**How to cite this article:** Bajwa NK, Jingarwar MM, Pathak A. Single Visit Apexification Procedure of a Traumatically Injured Tooth with a Novel Bioinductive Material (Biodentine). Int J Clin Pediatr Dent 2015;8(1):58-61.

## INTRODUCTION

Apexification is defined as a procedure used to induce a calcified barrier in a root with an open apex or the con-tinued apical development of an incomplete root in teeth with necrotic pulp. In more recent years, stress has been toward developing materials which do more than simply aim to replace lost tooth tissue but rather seek to induce its repair and regeneration. Mineral trioxide aggregate (MTA) was the first material to be used to induce apical third barrier in single visit apexification procedures. Biodentine cement is part of a new approach seeking to simplify clinical procedures.^1^

**Fig. 1:**
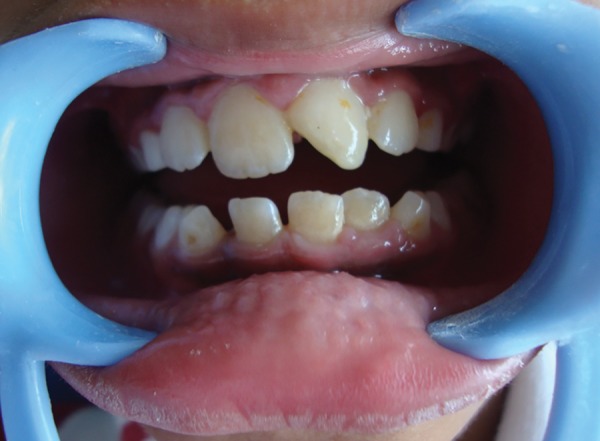
Preoperative view showing fractured 21

## CASE REPORT

**Fig. 2:**
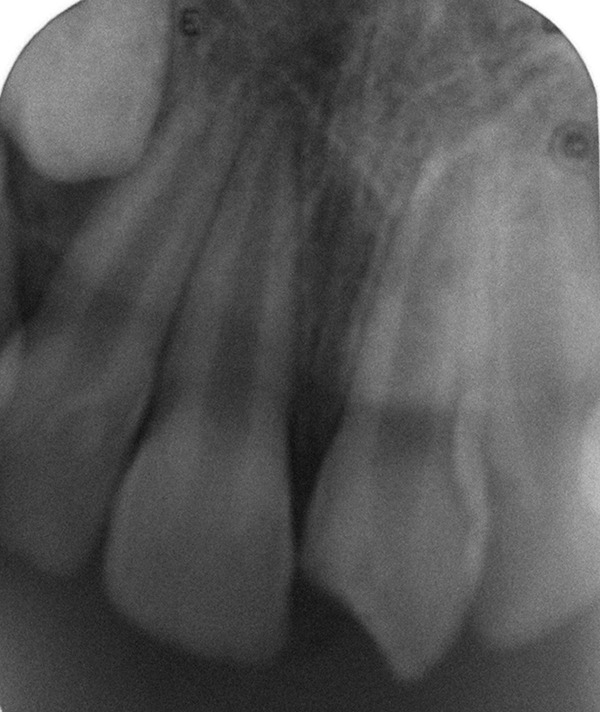
Preoperative radiograph showing oblique fracture line and arrested apical root development

**Fig. 3:**
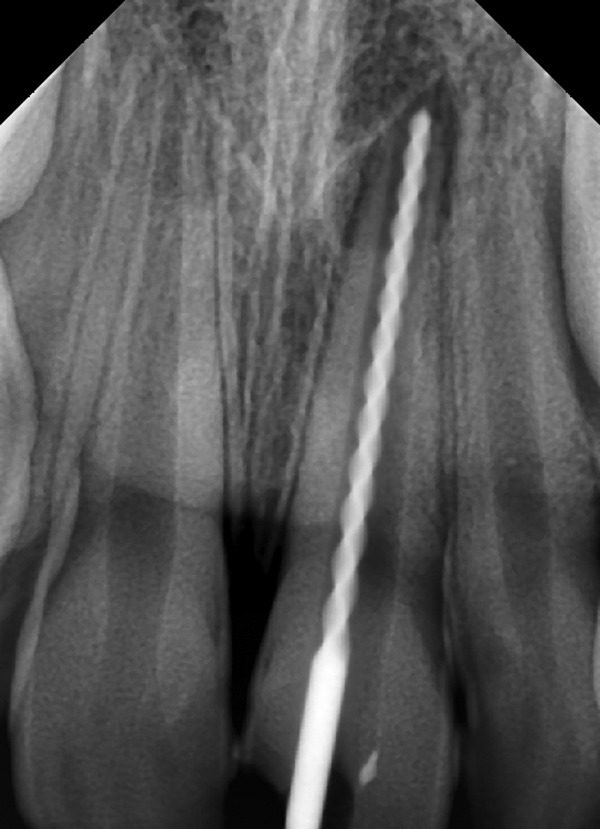
Working length determination

**Fig. 4:**
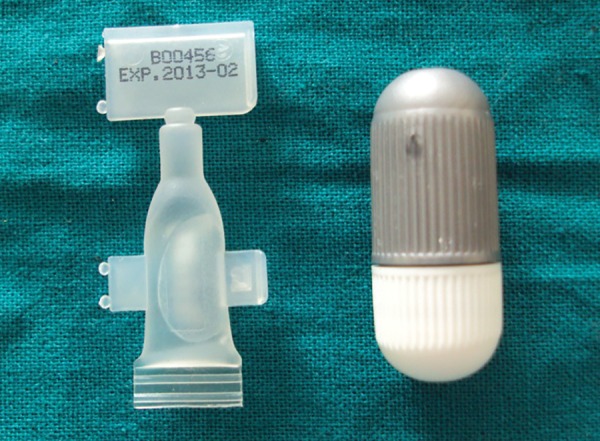
Biodentine available in the form of powder capsule and distilled water ampule

A 10 years old male patient presented to our department with dental trauma that had occurred 1 week back resulting in coronal fracture of the left upper central incisor (Fig. 1). Electrical pulp testing revealed positive results hence, indirect pulp capping was done and patient was recalled at regular intervals. No symptoms were reported by the patient but on clinical assessment, electrical pulp testing yielded negative results and radiographic assess-ment at the 6 months recall visit showed the apical root development of the tooth to be arrested (Fig. 2). It was, therefore, decided that apexification was indicated before endodontic treatment could be carried out for the affected tooth. The treatment plan was to form an artificial apical barrier after cleaning and decontamination of the canal with sodium chloride solution. Access cavity preparation was done for the tooth under rubber dam and working length was determined by the radiographic method (Fig. 3). Shaping was limited to the coronal third of the canal (with gates glidden drills) to facilitate direct instrument access to the foramen. Biodentine is an inorganic non-metallic compound presented in the form of a capsulated powder and a liquid twist cap bottle^2^ (Fig. 4). The capsule con-taining the powder was tapped and opened, followed by the addition of five drops of liquid from the single dose container to the capsule. The capsule was then closed and placed in an amalgamator for 30 seconds. The mix obtained is creamy in consistency, can be manipulated for 6 minutes and takes a further 6 minutes for setting.

**Fig. 5:**
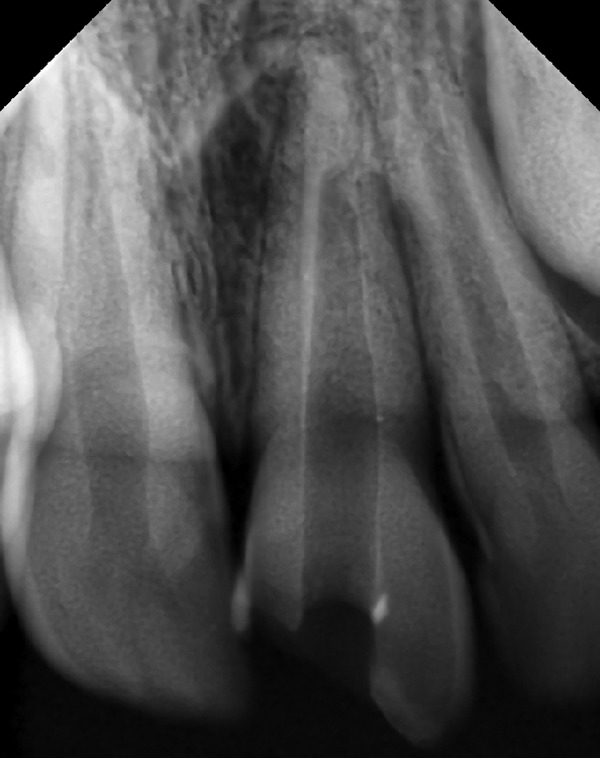
Apical barrier induction with Biodentine

**Fig. 6:**
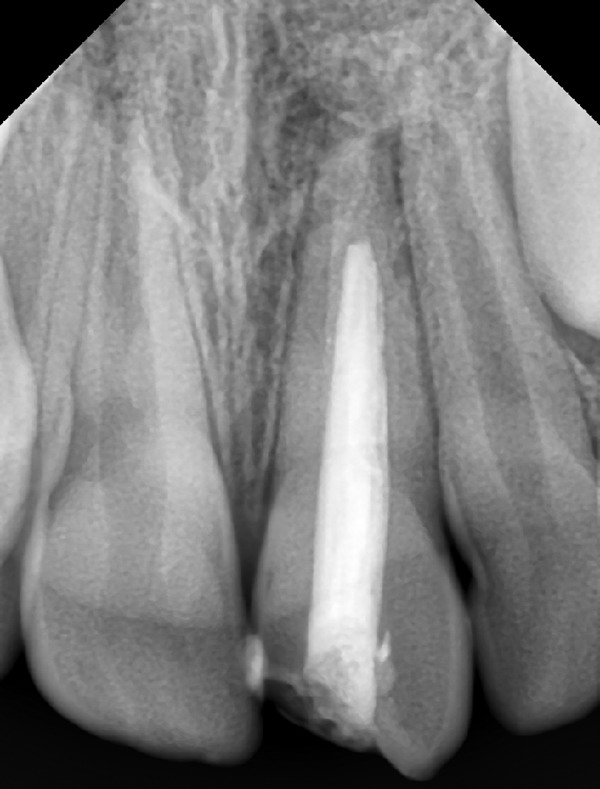
Intraoral periapical view showing obturation of the canal with gutta-percha

**Fig. 7:**
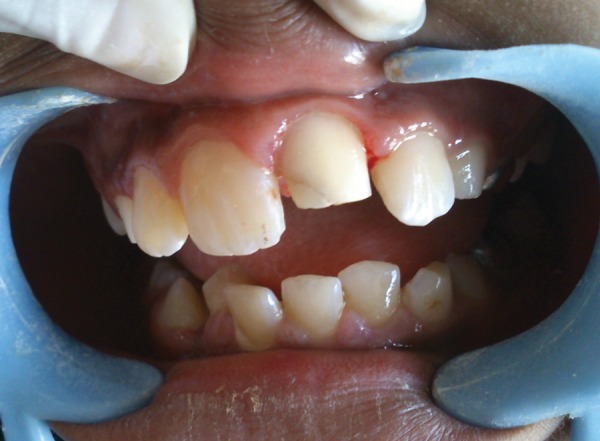
Intraoral view showing crown cutting for full coverage prosthesis

**Fig. 8:**
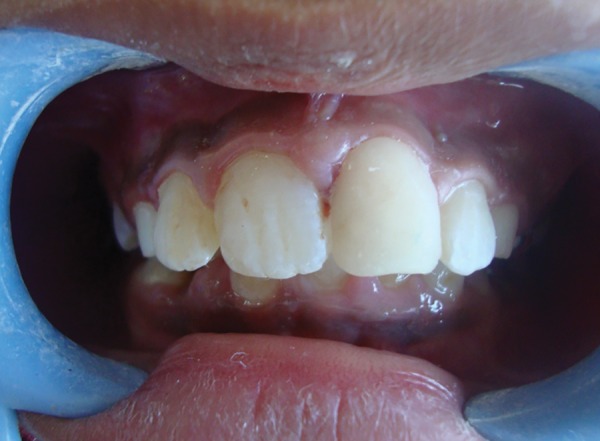
Intraoral view showing full coverage of all acrylic interim crown

The canal was filled with two increments of Bioden-tine with an amalgam carrier. The first increment of Biodentine was inserted into the canal using a reamer of the largest diameter fitting into the canal–ISO size 110. The material was then delicately pushed toward the apex with a root-canal plugger of ISO size 100. Several increments were similarly inserted and then condensed to form a plug of adequate thickness (> 4 mm). After verifying that the material was hard-set, the thickness of the apical barrier and adaptation of the material to the dentinal walls was confirmed radiographically (Fig. 5). Obturation of the canal was done with gutta-percha using the lateral condensation technique (Fig. 6). Post obtura-tion complete coverage prosthesis was planned for the tooth. Crown preparation was done (Fig. 7) and a full coverage acrylic crown was luted into place using resin-modified glass ionomer cement (Fig. 8). It will serve as a medium term solution for protection of the endodonti-cally treated tooth and esthetic restoration of the crown morphology as well.^1^

The patient was recalled after 1 month. History and clinical examination showed satisfactory healing, and an intraoral periapical view was taken which showed adequate periapical response as well.

## DISCUSSION

Proper radiographic and clinical assessment is very important in young permanent teeth which have undergone trauma and have been treated with vital pulp therapy. Pulpal sensibility testing at regular intervals can reveal the negative vitality status of a tooth which initially showed a positive response. Such, teeth will invariably have arrested apical root development. Such, teeth are an ideal indication for the procedure of apexification. Classically, apexification was performed by filling the canal with calcium hydroxide and iodoform mixture for a period of approximately 3 to 6 months. Bioactive materials like MTA and later on Biodentine were developed to form artificial apical barrier in a single visit so as to provide a positive stop for the obturating material.

Biodentine has a modified powder composition (Table 1), the addition of setting accelerators and softeners, and a new predosed capsule formulation for use in a mixing device, largely improved the physical properties of this material making it much more user-friendly.^3^

Tricalcium silicate mixes with the water component and leads to the formation of a hydrated calcium silicate gel (C-S-H) structure and calcium hydroxide.^4^ Crystallization results in the formation of CaCO_3_ crystals. The crystals of CaCO_3_ slowly fill in the porosities between the unreacted grains of cement over a period of approxi-mately 2 weeks until finally reaching a maximum.

The complete hydration reaction is as follows:

2(3CaO·SiO_2_) + 6H_2_O → 3CaO·2SiO_2_·3 H_2_O + 3Ca(OH)_2_

It is proposed that the micromechanical bond between dentin and Biodentine is formed via crystal growth within the dentin tubules, leading to a micromechanical anchor or possible ion exchanges between the cement and dentinal tissues.

At the material-dentin interface, Biodentine reportedly forms a tag-like crystalline structure within the dentinal tubules. Further more, a Caand Sirich interfacial layer is observed under magnification. This layer causes chemical and structural modification of the surrounding dentin, which may result in higher acid resistance and physical strength. Mechanical properties of this material are superior in terms of compressive strength (220 MPa), flexural strength (34 MPa), elastic modulus (22.000 MPa) and Vickers hardness (60 HV) (Table 2). This novel mate-rial, hence, is biologically active, has improved physical, chemical and mechanical properties and, therefore, can have a multitude of potential clinical indications.

## CONCLUSION

This new calcium-based cement with its purported biocompatible and bioactive properties aims to regenerate damaged dental tissues and represents a promising evolution of the MTA technology.^5^ The evidence-based research for this material’s properties is, however, limited at present. Currently, experimental *in vitro*, *in vivo* and *ex vivo* results have so far substantiated and the manufacturers claims that the material is bioactive and biocompatible. However, the number of studies are small and half have used animal models, which offer an imperfect comparison to human dentinopulpal responses. The clinical *in vivo* trials are still ongoing and further trials will be needed to determine whether the *in vitro* results will translate into clinical practice. Importantly, independent case reports, clinical trials and randomized control trials are lacking and necessary, if this material is to become a permanent fixture within the clinician’s armamentarium.^5^

**Table 1:** **Table 1:** The composition and function of various components of Biodentine

*Powder (1 gm)*		*Function*		*Liquid (200 ml)*		*Function*	
Tricalcium silicate (3CaOSiO_2_)		Main component of the powder. It regulates the setting reaction		Calcium chloride (CaCl_2_.2H_2_O)		Acts as an accelerator	
Calcium carbonate (CaCO_3_)		Acts similar to a filler		Water reducing agent			
Zirconium dioxide (ZrO_2_)		Provides radiopacity to the cement				Reduces the viscosity of the cement to achieve workability, while reducing the water content	

**Table 2:** **Table 2:** Comparison of mechanical properties of Biodentine with dentin and other restorative materials

*Materials*		*Compressive strength (MPa)*		*Flexural strength (MPa)*		*E-modulus (MPa)*		*Vickers hardness (VH)*	
Biodentine^™^		220		34		22.000		60	
Dentin		200-350		20		15.00-20.000		60-90	
GIC		140-180		10-21		5.000-11.850		60	
Composite		290-400		100-145		12.000-16.000		70-130	

## CLINICAL SIGNIFICANCE

Biodentine which is a new biologically active cement can be used as a dentin replacement and an efficient alternative to the conventional apexification materials which were hitherto recommended.
